# Deciphering the Link Between ER^UPR^ Signaling and MicroRNA in Pathogenesis of Alzheimer’s Disease

**DOI:** 10.3389/fnagi.2022.880167

**Published:** 2022-05-09

**Authors:** Nusrath Yasmeen, Manali Datta, Vikram Kumar, Fahad S. Alshehri, Atiah H. Almalki, Shafiul Haque

**Affiliations:** ^1^Amity Institute of Biotechnology, Amity University Rajasthan, Jaipur, India; ^2^Department of Pharmacology and Toxicology, College of Pharmacy, Umm Al-Qura University, Makkah, Saudi Arabia; ^3^Department of Pharmaceutical Chemistry, College of Pharmacy, Taif University, Taif, Saudi Arabia; ^4^Addiction and Neuroscience Research Unit, College of Pharmacy, Taif University, Taif, Saudi Arabia; ^5^Research and Scientific Studies Unit, College of Nursing and Allied Health Sciences, Jazan University, Jazan, Saudi Arabia; ^6^Faculty of Medicine, Bursa Uludağ University, Bursa, Turkey

**Keywords:** ER stress, unfolded protein response (UPR), microRNA, Alzheimer’s disease, neurodegeneration

## Abstract

Alzheimer’s disease (AD) is a neurodegenerative proteinopathic disease. The deposits of misfolded Amyloid β and Tau proteins in the brain of patients with AD suggest an imbalance in endoplasmic reticulum (ER) proteostasis. ER stress is due to accumulation of aberrant proteins in the ER lumen, which then leads to activation of three sensor protein pathways that ultimately evokes the adaptive mechanism of the unfolded protein response (UPR). The UPR mechanism operates *via* adaptive UPR and the apoptotic UPR. Adaptive UPR tries to restore imbalance in ER hemostasis by decreasing protein production, enhanced chaperone involvement to restore protein folding, misfolded protein decay by proteasome, and suppression of ribosomal translation ultimately relieving the excessive protein load in the ER. Subsequently, apoptotic UPR activated under severe ER stress conditions triggers cell death. MicroRNAs (miRNAs) are small non-coding protein causing dysregulated translational of mRNAs in a sequential manner. They are considered to be critical elements in the maintenance of numerous cellular activities, hemostasis, and developmental processes. Therefore, upregulation or downregulation of miRNA expression is implicated in several pathogenic processes. Evidence from scientific studies suggest a strong correlation between ER^UPR^ signaling and miRNA dysregulation but the research done is still dormant. In this review, we summarized the cross-talk between ER stress, and the UPR signaling processes and their role in AD pathology by scrutinizing and collecting information from original research and review articles.

## Introduction

The endoplasmic reticulum (ER) is a cellular organelle with an imperative role in the processes of folding, modification, and trafficking of nascent proteins (Plácido et al., 2014). ER dysfunction leads to an imbalance in the proteostasis that results in accumulation of abnormal (unfolded/misfolded) proteins, consequently, such proteins trigger a stress response called ER stress (Scheper and Hoozemans, 2015; Rahman et al., 2017). ER dysfunctions/ER stress is considered to be involved in several pathological conditions like metabolic disorders, such as diabetes, neurodegenerative diseases, cancer, atherosclerosis, lung, and renal diseases (Lindholm et al., 2017). ER stress in turn triggers unfolded protein response (UPR), an adaptive rescuer mechanism, through upregulation of molecular chaperones (Fu and Gao, 2014; Alshareef et al., 2021). The UPR acts as *two-edged sword* it can either exhibit cytoprotective action or cytotoxic action. Cytoprotection is exhibited through the revival of proteostatic balance, and cellular adaptations essential for ER protein folding. However, prolonged ER stress-related perturbations might enhance UPR led cytotoxicity and hence apoptosis (Gardner et al., 2013).

Proteostatic defects augment the accretion of misfolded protein, to which neuronal cells are sensitive. Consequently, they survive and evade toxicity by utilizing the ER-induced UPR mechanism (Remondelli and Renna, 2017). Misfolded protein aggregates may cause neuronal toxicity, which is regarded as the crucial step in the pathogenesis of neurodegenerative diseases (Spires-Jones et al., 2017). Alzheimer’s disease (AD) is a type of misfolded-protein disease. AD is an age-related neurodegenerative disease regarded as the most common cause of dementia (Abduljaleel et al., 2014; Scheper and Hoozemans, 2015; Rahman et al., 2018). The prominent neuropathological features of AD include Neuritic plaque (NP) besides neurofibrillary tangle (NFT). NPs are extracellular aggregates of amyloid-β (Aβ) peptides (Labban et al., 2021). Furthermore, dystrophic neurites and neuropil threads along with interneuronal aggregates of hyperphosphorylated Tau proteins in neurofibrillary tangles are other hallmark features of AD (Tanprasertsuk et al., 2019). Katayama et al., in their study, reported that mutation in presenilin-1 (PS1), and induction of high-mobility group A1a (HMGA1a) protein by Presenilin-2 (PS2) isoform, i.e., PS2V, along with the activation of Caspase-4 are all responsible for the neuronal cell death in AD. Eventually, this is attributed to hyperactivity of the protein translation mechanism accompanied by Aβ accumulation in neurons all of which lead to aberrant ER stress (Katayama et al., 2004; Wu Q. et al., 2016; Hashimoto and Saido, 2018).

MicroRNAs (miRNAs) are non-coding 19–25 nucleotides long, single-stranded protein molecules that are endogenously synthesized, evolutionarily conserved (Rahman et al., 2021; Yasmeen et al., 2021). miRNA mediates post-translational gene regulation either by promoting mRNA decay or repressing protein translation (Wang M. et al., 2019). The role of miRNAs is imperative in several cellular processes, such as cellular proliferation, cell differentiation, apoptosis, and in neural cell biology. Local protein synthesis to crucial to maintain neuronal function and synaptic plasticity. The existence of several hundreds of translationally silent mRNAs in axons their activation *via* stimuli leads to their subsequent protein synthesis specific to that cellular process (Kim and Jung, 2015). miRNAs are located in cell soma, dendrites, and synaptosomes, they control GluA1 or GluA2 subunits of AMPA-type glutamate receptor (AMPAR) along with GABA_A_Rs (α1 and γ2) crucial for synaptic coordination and long-term potentiation. Hence, miRNAs are regarded as key regulators of local protein synthesis during synaptic plasticity. In short, neuronal protein translation is also regulated by miRNAs (Smalheiser and Lugli, 2009; Rajgor et al., 2021). Not only this, but they are also implicated in several pathological processes, such as cancer, diabetes, and neurodegenerative diseases (Grasso et al., 2014). The role of miRNAs in regulating ER stress is the foundation to unravel following several studies. However, significant contributions are required to understand the mechanism involved behind this. Numerous revelations are being made in recent times, regarding the influence of miRNA dysregulation in neuronal disease. Several miRNA families for instance miR-485 were found to be dysregulated in AD and other neurological diseases. In this review, we summarize the connection between miRNAs and ER^UPR^ signaling pathways that play a vital role in AD.

## ER^UPR^ Signaling

**FIGURE 1:**
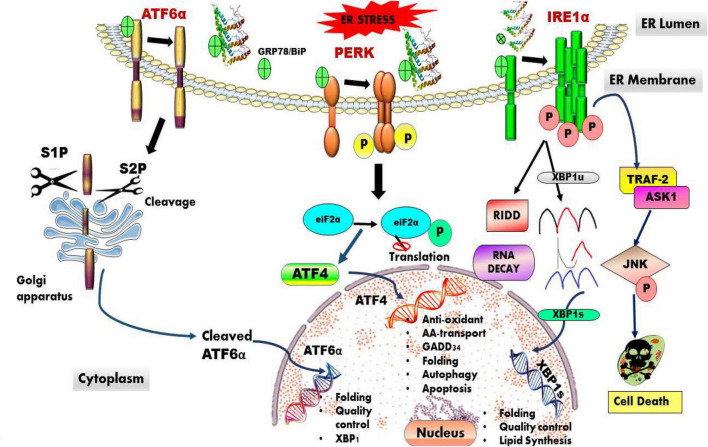
Overview of ER-induced UPR stress signaling pathway. The UPR is an adaptive mechanism activated in response to ER stress. The three UPR stress sensing proteins that are activated are IRE1, PERK, and ATF6, which work independently with an aim to maintain protestatic balance *via* decreasing the load of misfolded proteins and restoring ER protein homeostasis.

The ER is a membrane-bound organelle that plays an important role in the synthesis, folding, maturation, proper trafficking, quality control, degradation, and post-translational modifications of nascent proteins. It is also involved in several other cellular events, such as calcium homeostasis, cholesterol homeostasis in endosomes, and in cytoplasmic streaming (Almanza et al., 2019). ER quality control helps in preserving proteostasis. ER-associated degradation (ERAD) is a process that helps in disposing of terminally misfolded proteins through ubiquitin–proteasome system (Oku et al., 2021). When the misfolded proteins are overloaded the prototypical ER-stress sensors located at the ER membrane activate UPR. The UPR activation is mediated by an intricate network of three main signaling pathways: inositol-requiring transmembrane kinase/endonucleases (IRE1α and -β), protein kinase RNA (PKR)-like endoplasmic reticulum kinase (PERK), and the activating transcription factor-6 (ATF6α and -β) (Dufey et al., 2014). Under normal physiological conditions, these stress-sensor proteins bind to BiP/GRP78 an ER-resident chaperone forming a complex, which remains dormant (Hoozemans et al., 2012). Whereas, following ER stress this complex dissociates and individual proteins are activated and influence downstream signaling. The signaling pathways involved in UPR are depicted in Figure 1.

### IRE1 Pathway

Inositol-requiring transmembrane kinase/endonucleases 1 (IRE1) is a highly conserved, type I transmembrane protein. In ER-stress conditions, IRE1 self-associates during which BiP/GRP78 chaperone is displaced by Hsp47, from the complex called IRE1 UPRosome and promotes oligomerization (Almanza et al., 2019). The activation of cytosolic kinase domain and IRE1 RNase domain is through conformational change and autophosphorylation. Activation of IRE1 further activates UPR downstream signaling *via* the splicing of X-box binding protein 1 (Xbp-1) mRNA, which is a basic leucine zipper (bZIP) transcription factor (Gerakis and Hetz, 2018). Spliced XBP1 (sXBP1) mRNA mediates transactivation of genes, such as GRP78/BiP essential for proteostasis, lipid biosynthesis, redox metabolism, and glucose metabolism. Furthermore, the activated RNase domain of IRE1 leads to activation of regulated IRE1-dependent decay (RIDD). RIDD pathway activation degrades mRNAs and miRNAs, decreasing the load of nascent proteins entering ER. Hence, essential for preserving ER homeostasis and cell survival (Siwecka et al., 2021). Under ER stress conditions, IRE1α induces TRAF 2 and ASK1 [tumor necrosis factor (TNF) receptor-associated factor 2; apoptosis signal-regulating kinase 1], respectively. Subsequently, the IRE1α-TRAF2-ASK1 complex formed activates several other pathways, such as JNK (c-Jun N-terminal kinase), MAPK (p38 mitogen-activated protein kinase), and procaspase 12 pathways, which ultimately lead to apoptosis. Hence, IRE1α pathway is regarded as the main trigger of apoptosis (Chen and Brandizzi, 2013; Wu J. et al., 2016; Junjappa et al., 2018).

### PERK Pathway

Protein kinase RNA (PKR)-like endoplasmic reticulum kinase is a kinase protein that is ubiquitously expressed lodges ER luminal domain and a cytoplasmic kinase domain. Under ER-stress conditions, the PERK pathway is activated when BiP/GRP78 chaperone and PERK complex dissociates, which leads to oligomerization, additionally, its cytosolic kinase domain is subjected to trans-auto phosphorylation. PERK activation phosphorylates eukaryotic translation initiation factor-2α(eIF2α) on serine 51 causing downstream signaling of eIF2α (Wang et al., 2018). Phosphorylation of eIF2α is involved in regulation of protein synthesis, also inhibits eIF2B activity, which blocks protein translation and hence reduces the protein load. eIF2α phosphorylation translates mRNA having short upstream open reading frames (uORFs) that activates ATF4, which in turn induces GADD34 (growth arrest and DNA damage-inducible34) and C/EBP homologous protein (CHOP) (Pakos-Zebrucka et al., 2016). GADD34 is a regulatory subunit of protein phosphatase (PP1C) complex, it acts as a negative feedback mechanism, as it dephosphorylates eIF2α in the PERK pathway, while CHOP is a proapoptotic factor. Overall PERK/ATF4 signaling is involved in redox homeostasis, protein folding, amino acid biosynthesis, autophagy, and apoptosis. PERK pathway is prosurvival and transitions to proapoptotic under critical ER-stress conditions (Gardner et al., 2013).

### ATF6 Pathway

Activating transcription factor 6 a type II transmembrane protein has stress sensor carboxy-terminal luminal domain and an amino-terminal domain, which is a bZip transcription factor (Stauffer et al., 2020). ER-stress conditions activate ATF6, which translocate from the ER to the Golgi, where it is cleaved sequentially by the site-1 and site-2 proteases (S1Pand S2P) releasing the cytosolic amino terminus ATF6-N fragment. ATF6-N later binds to cis-acting ER stress response elements (ERSE) in the nucleus. ATF6 up-regulates gene expression of foldases, chaperones, XBP-1, and ERAD pathway also facilitates cytoprotection, suppressing the UPR-induced apoptotic program (Ibe et al., 2021). Figure 1 depicts the various pathways activated in ER proteotoxic stress conditions.

## MicroRNAs Implicated in Alzheimer’s Disease

Alzheimer’s disease is a neurodegenerative disease with dual proteinopathy characterized by deposits of neuritic ß-amyloid (Aß) plaques and deposits of hyper-phosphorylated, microtubule stabilizing protein called tau protein in NFTs (Bejanin et al., 2017; Sultan et al., 2020). Neuropsychiatric symptoms (NPSs) are prevalent hallmark symptoms in patients with AD. Furthermore, AD is characterized by impairment in cognitive, behavioral, and functional domains with symptoms, such as depression, apathy, cognitive decline, delusion, motor disturbance, and agility (Chen M. L. et al., 2021). miRNAs exhibit crucial role in the pathogenesis of several neurodegenerative diseases, such as AD, they may be either upregulated or downregulated in AD. The onset of AD begins with synaptic dysfunction, which is accompanied by the dysregulation of several miRNAs. miRNAs serve as a promising biomarker to monitor initial, pre-symptomatic phases of AD pathogenesis. Evidence from previous studies suggests that several miRNAs are implicated in the AD pathogenesis by dysregulating functions of Aβ production, Cofilin, APP, BACE1, and Tau phosphorylation levels. There are numerous miRNAs implicated in the pathogenesis of AD some of them are elaborated in the following Table 1.

**TABLE 1:** Depicts several microRNAs dysregulated in AD.

microRNA	Up/Down regulation	Description	Experimental model	Potential targets	References
miR-34c	Up regulated	Causes synaptic and memory deficits by targeting SYT1 through ROS-JNK-p53 pathway	HT-22 cells; SAMP8 mice and patients	Synaptotagmin 1 (SYT1)	Shi et al., 2020
miR-455-5p	Up regulated	Led to synaptic and memory deficits in AD	APP/PS1 mice	Cytoplasmic polyadenylation element-binding 1 (CPEB1)	Xiao et al., 2021
miR-361-3p	Up regulated	inhibits β-amyloid deposition and ameliorated AD progression	SH-SY5Y cell; APP/PS1 mice	BACE1	Ji Y. et al., 2019
miR-181a	Up regulated	mediates Aβ-induced synaptotoxicity; Leads to loss of functional synapses and cognitive impairment	C57BL6/J mice and 3xTg-AD	GluA2	Rodriguez-Ortiz et al., 2020
miR-200b/c	Up regulated	Exhibit defensive role against Aβ-induced toxicity by activating insulin signaling pathway	Tg2576 transgenic Mice	Ribosomal protein S6 kinase B1 (S6K1)	Higaki et al., 2018
miR-22-3p	Up regulated	reduced Aβ deposit reduce AD progression	AD mice model	Mitogen-activated protein kinase 14 (MAPK14)	Ji Q. et al., 2019
miR-26b	Up regulated	Activates Cell Cycle, Hyper phosphorylation of Tau and Apoptosis in Neurons	MCI and AD brains	IGF-1; Cdk5; Retinoblastoma protein (Rb1)	Absalon et al., 2013
miR-9	Up/down regulated	Helps in hyperphosphorylation of Tau		Fibroblast growth factor receptor 1 (FGFR1), NFkB and sirtuin 1 (SIRT1)	Delay et al., 2012; Almalki et al., 2021
miR-485-3p	Up regulated	Aβ plaque deposition, tau phosphorylation,	Transgenic mice and AD patients’	Synaptophysin	Koh et al., 2021
miR-200a-3p	Up regulated	inhibit cell apoptosis; reduced the production of Aβ1-42; neuroprotective effects	APP/PS1 and SAMP8 mice; AD patients	BACE1 and protein kinase cAMP-activated catalytic subunit beta (PRKACB)	Wang L. et al., 2019
miR-409-5p	Down regulated	impairs neurite outgrowth, decreases neuronal viability, and accelerates the progression of Aβ1 - 42-induced pathologies	APP/PS1 mice	Pleckstrin (Plek)	Guo et al., 2019
miR-106b	Down regulated	APP expression regulation; Cell cycle regulation; apoptosis and autophagy	SH-SY5Y cells	Rb1, p2; APP; p73; p62; Fyn	Liu et al., 2016
miR-384	Down regulated	decreased miR-384 levels might cause upregulation of APP and lead to progression of AD	SH-SY5Y cells; Patients with MCI and DAT	APP and BACE-1	Liu et al., 2014
miR-135a-5p	Down regulated	loss of miR-135a-5p, aberrant Rock2 activation…reduction of Foxd3 are induced by Tau and are associated with memory impairment and synaptic disorder	APP/PS1 mice and P301S	Foxd3	Zheng et al., 2021
miR-331-3p and miR-9-5p	down-regulated ….early-stage up-regulated late-stage of AD	Affected autophagy	APPswe/PS1dE9 mice	Sequestosome 1 (Sqstm1) and Optineurin (Optn)	Chen Y. et al., 2021
miR-335-5p	Down regulated	Aβ accumulation and AD progression, activate JNK pathway	APP/PS1 transgenic mice	c-jun-N-terminal kinase 3 (JNK3)	Wang et al., 2020
miR-16-5p	Up regulated	neuronal cell apoptosis in AD	5xFAD mice; SH-SY5Y cells	B cell lymphoma-2 (BCL-2)	Kim et al., 2020
miR-214-5p	Down regulated	Involved with hippocampal neuronal apoptosis, cognitive impairment and oxidative stress	APPswe/PS1dE9 mice	SUZ12	Hu et al., 2021
miR-124	Down regulated	alleviated Aβ-induced viability; decreased apoptosis	SH-SY5Y cells.	BACE1	An et al., 2017
miR-29a/b-1	Down regulated	AD progression	sporadic AD patients	BACE1	Hébert et al., 2008
miR-339-5p	Down regulated	AD progression	AD patients	BACE1	Long et al., 2014
miR-149	Down regulated	AD Progression	SH-SY5Y cells	BACE1	Du et al., 2021

## Connection Between ER^UPR^ Signaling Pathways and miRNAs in Alzheimer’s Disease

**FIGURE 2:**
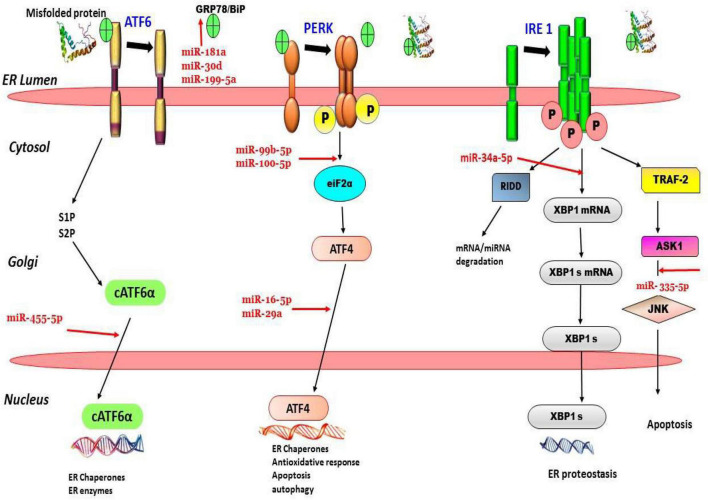
ER stress, UPR, and microRNA relation in Alzheimer’s disease. For instance, evidence suggests that, miR-199-5a, miR-30a and miR-181 suppress GRP78 expression. miR-34a-5p downregulates XBP-1 expression in. miR-335 is involved in activation of JNK pathway leading to apoptosis, etc., can be seen in the figure.

MiRNAs are proven to be the most efficient modulators of several cellular pathways. They also play a regulatory role in the major ER-stress sensor pathways like IRE1, ATF6, and PERK pathways. miRNAs help in modulation of the cellular stress response, by regulating UPR key component levels. Krammes et al., 2020 reported that miR-34a-5p plays a vital role in the regulation of the IRE1α branch. Its overexpression targets *BIP*, *IRE1*α, and *XBP1* genes and subsequently leads to a reduction in IRE1α and XBP1s thereby modulating UPR signaling. In another study, it was reported that protection against Aβ-induced injury in SH-SY5Y cells can be enhanced by miR-34a upregulation and IRE1α inhibition (Li et al., 2019). It is also suggested that a plausible link exists between miR expression levels and Aβ-induced ER stress, they reported that upregulation of miR-200c induced by Aβ deposition is mediated *via* ER stress pathways, such as PERK and eIF2 α pathways, due to the activation of as XBP1, ATF4, and ATF6 transcriptional factors (Wu J. et al., 2016). The findings of a recent study reported significant alterations in the levels of two miR-99b-5p and miR100-5p *via* Aβ-induced ER-stress modulation along with alteration in the mTOR pathway. This proved the correlation between the ER stress–miRNAs–mTOR” axis in AD pathogenesis (Ye et al., 2015). Figure 2 depicts ER stress and microRNA relation in AD. From the above studies, it is evident that a significant relationship exists between ER stress pathways—miRNAs axis but the exact mechanism still remains elusive and needs further elaborative research to gather substantial evidence.

### Therapeutic Approaches Against Alzheimer’s Disease Involving the ER^UPR^ Signaling Pathway and MicroRNA

Alzheimer’s disease pathobiology is complex and still elusive. Although, NFT, Aβ, and tau proteins are regarded as the markers of this disease, treatment approaches are dormant. The first drug ever known to treat cognitive symptoms of AD is Tacrine, which is currently withdrawn due to toxicity issues. Later many drugs targeting cholinesterase, NMDA receptors were introduced which could only alleviate symptoms. The year 2021 saw a major breakthrough with the approval of drug, Aducanumab, an IgG1 monoclonal antibody targeted to treat the underlying cause of disease (Riscado et al., 2021). Another drug Verubecestat (MK-8931) targeting beta-secretase 1 (BACE1), was supposedly being investigated to treat AD; however, this was terminated due to no promising results (Kennedy et al., 2016). miRNAs are identified as efficient diagnostic biomarkers for AD, they target multiple genes and multiple pathologic pathways in one stance. Hence, miRNAs are regarded as potential therapeutic targets for several diseases, including AD. However, there are several limitations to develop miRNA-based therapeutic agents, like difficulty in identification of the functional target gene, factors influencing variations in miRNA expression, decay kinetics of miRNA in body fluids, passage across BBB, the delivery options of miRNA in their stable form, toxicity, and immune response (Madadi et al., 2019). Scientists around the world are working tremendously to overcome these hurdles. Currently, encouraging developments are seen targeting diseases, using miRNA mimics, miRNA sponges/decoys, antagomiRs, miRNA antisense nucleotides, antagoNAT and miRNA-based oligonucleotides (Bajan and Hutvagner, 2020). Also, delivery of these miRNA, which is a hurdle, is being worked on using novel targeted delivery approaches, such as conjugated nanoparticle, intra-nasal administration, intracerebroventricular infusion, lipid, and viral vectors (Fu et al., 2019). The first drug developed using miRNA targeting is Miravirsen, miRNA oligonucleotide to treat hepatitis C, which is currently in phase II clinical trials (Baek et al., 2014). Despite such incredible efforts by the scientific fraternity no approved disease-modifying miRNA-based AD therapies 0 exists, no drug have been FDA approved as yet. However, several miRNA-based drugs have managed to reach phase I and phase II clinical trials. These lacunae need to be addressed as early as possible so as to enable AD treatment with novel therapeutic approaches.

## Conclusion and Future Perspectives

The endoplasmic reticulum has a very crucial role in several cellular processes with a focus on protein synthesis and transport, proteostasis, redox homeostasis, autophagy, and cellular apoptosis, supposedly disrupted in aberrant conditions ameliorating AD progression. Numerous studies have shown the role of regulatory non-coding proteins miRNA in several pathologies, such as AD. This article reviews the complex, interconnection between non-coding miRNA networks involved in the regulation of ER stress *via* UPR activation. Hundreds of miRNAs have been discovered and still there is scope for the discovery of miRNAs that are directly involved in altering and regulating ER-induced UPR stress pathways. miRNAs exhibit diversity in their actions, advancements in molecular and RNA sequencing technologies have provided significant insights into the dysregulation in miRNAs in AD and helped us unravel their role as potential diagnostic biomarkers and therapeutic agents. An elaborative analysis of individual miRNAs or clusters of miRNA families targeting ER^UPR^ or ER proteostasis along with miRNA quantification methods must be carried out to precisely know the miRNAs that can serve as excellent biomarkers and therapeutic targets to overcome the disease burden of AD.

## Author Contributions

NY, MD, VK, and FA conceptualized the idea of mini-review and were responsible for the entire manuscript in its final form. AA and SH checked the complete manuscript in terms of technical and scientific integrity. All authors contributed to manuscript writing, revision, read, and approved the submitted version.

## Conflict of Interest

The authors declare that the research was conducted in the absence of any commercial or financial relationships that could be construed as a potential conflict of interest.

## Publisher’s Note

All claims expressed in this article are solely those of the authors and do not necessarily represent those of their affiliated organizations, or those of the publisher, the editors and the reviewers. Any product that may be evaluated in this article, or claim that may be made by its manufacturer, is not guaranteed or endorsed by the publisher.
